# Supplementary material for: Mechanism of B-box 2 domain-mediated higher-order assembly of the retroviral restriction factor TRIM5α
Source: eLife. 2016 Jun 2;5:e16309. doi: 10.7554/eLife.16309 (PMC4936894; doi:10.7554/eLife.16309)
Supplement: Supplementary file 1. — DOI: http://dx.doi.org/10.7554/eLife.16309.023 [file elife-16309-supp1.docx]

**Supplementary file 1A.**

Crystallization conditions for Bcc miniTRIM.

| **Spacegroup** | **Oligomeric State** | **Well Solution** | **Protein:Precipitant Drop Ratio** | **Resolution (Å)** | **Refined?** |
| --- | --- | --- | --- | --- | --- |
| C2 | Dimer | 0.1 M Tris pH 7.0  0.2 M NaCl  28% PEG 3350 | 1:1 | 1.9 | Yes |
| P6222 | Dimer | 0.1 M HEPES pH 7.5  20% PEG 4000  10% isopropanol | 3:1 | 1.9 | No |
| P1 | Dimer | 0.1 M Na Acetate pH 4.6  0.2 M Ca Acetate  23% PEG 3350 | 1:1 | 2.3 | Yes |
| R32 | Dimer | 0.1 M Tris pH 9.0  0.4 M Ca Acetate  25% PEG 4000 | 1:1 | 3.5 | No |
| P212121 | Trimer | 0.1 M HEPES pH 6.8  0.2 M NaCl  23% PEG 3000 | 2:1 | 3.3 | Yes |
| P21 (twinned) | Trimer | 0.1 M HEPES pH 6.6  0.25 M NaCl  25% PEG 3350 | 2:1 | 2.8 | No |

**Supplementary file 1B.**

Average root mean square deviations over equivalent Cα atoms from superpositions of the miniTRIM monomer structures with 4TN3 (Goldstone et al., 2014).

| **MiniTRIM monomer** | **B-box/mini-cc*** | **B-box only*** | **Mini-cc only*** |
| --- | --- | --- | --- |
| P212121 trimer chain A | 1.636 | 0.712 | 1.322 |
| P212121 trimer chain B | 1.675 | 0.779 | 1.359 |
| P212121 trimer chain C | 1.621 | 0.728 | 1.348 |
| P212121 trimer chain D | 1.541 | 0.774 | 1.329 |
| P212121 trimer chain F | 1.676 | 0.774 | 1.358 |
| P212121 trimer chain K | 1.537 | 0.777 | 1.322 |
| C2 dimer chain A | 1.735 | 0.901 | 1.762 |
| C2 dimer chain D | 1.418 | 0.838 | 1.313 |
| P1 dimer chain E | 1.184 | 0.591 | 1.070 |
| P1 dimer chain F | 0.931 | 0.564 | 0.960 |
| P1 dimer chain H | 1.098 | 0.589 | 1.211 |
| P1 dimer chain L | 1.141 | 0.579 | 1.232 |
| Average | 1.433 | 0.717 | 1.299 |
| Standard deviation | 0.273 | 0.112 | 0.192 |

*Residue ranges used: B-box = 95-132, mini-cc = 133-159 + 226-259
